# Irradiated fibroblasts increase interleukin-6 expression and induce migration of head and neck squamous cell carcinoma

**DOI:** 10.1371/journal.pone.0262549

**Published:** 2022-01-28

**Authors:** Shinsuke Suzuki, Satoshi Toyoma, Yohei Kawasaki, Takechiyo Yamada

**Affiliations:** Department of Otorhinolaryngology & Head and Neck Surgery, Akita University Graduate School of Medicine, Akita, Japan; King Faisal Specialist Hospital and Research Center, SAUDI ARABIA

## Abstract

**Background:**

Cytotoxic effects of radiation play an important role in the treatment of head and neck cancer. However, irradiation is known to lead to the migration of various cancer cells, including those of head and neck cancer. Recently, fibroblasts in the cancer microenvironment have been reported to be involved in this mechanism. Nevertheless, the mechanism underlying migration of head and neck cancer cells remains unclear. Herein, we aimed to elucidate this migration mechanism induced by irradiation in terms of the interaction of head and neck cancer cells with fibroblasts.

**Methods:**

We used the head and neck squamous cell carcinoma (HNSCC) cell lines SAS and FaDu as well as fibroblast cell lines. These cells were irradiated and their viability was compared. In fibroblasts, changes in interleukin-6 (IL-6) secretion caused by irradiation were measured by enzyme-linked immunosorbent assay (ELISA). The cell migration ability of cancer cells was evaluated via a migration assay using a semipermeable membrane. HNSCC cells were cocultured with irradiated and nonirradiated fibroblasts, and their migration ability under each condition was compared. We also examined the effect of IL-6 on the migration of HNSCC cells. Furthermore, to investigate the effect of fibroblast-derived IL-6 on the migration ability of HNSCC cells, we conducted a coculture study using IL-6 neutralizing antibody.

**Results:**

Irradiation reduced the survival of HNSCC cells, whereas fibroblasts were resistant to irradiation. Irradiation also increased IL-6 secretion by fibroblasts. Migration of HNSCC cells was enhanced by coculture with fibroblasts and further enhanced by coculture with irradiated fibroblasts. We also confirmed that the migration of HNSCC cells was induced by IL-6. The enhanced migration of cancer cells caused by coculturing with fibroblasts was canceled by the IL-6 neutralizing antibody.

**Conclusion:**

These results show that fibroblasts survive irradiation and induce the migration ability of HNSCC cells through increased secretion of IL-6.

## Introduction

Radiotherapy plays an important role in the treatment of head and neck cancer; indeed, it has been used at every stage of treatment [1, 2]. However, although irradiation has cytotoxic effects on cancer cells, it reportedly promotes tumorigenicity, and especially migration of cancer cells. This phenomenon results in increased local invasion, distant metastasis, and therefore poor prognosis for patients with various cancers including head and neck cancer [3, 4]. Thus, the conflicting effects of irradiation on cancer cells are a major challenge to be overcome in cancer treatment.

In recent years, it has been found that irradiation induces tumorigenic effects not only in cancer cells but also the cancer microenvironment. This alteration in the cancer microenvironment is reportedly involved in the recurrence and metastasis of cancer after radiotherapy [5, 6]. Mechanistically, the importance of various cytokines and growth factors in mediating the interaction between cancer cells and supporting cells, such as fibroblasts, in the cancer microenvironment has been confirmed [7–9]. In particular, interleukin-6 (IL-6) is known to be involved in the interaction between fibroblasts and cancer cells in the irradiated cancer microenvironment, and its role has attracted attention [10, 11].

To date, few studies have been conducted on irradiation-induced tumor progression in head and neck cancer; thus, much remains to be elucidated. The purpose of this study was therefore to elucidate the mechanism of irradiation-induced head and neck cancer cell migration in terms of the interaction of these cells with fibroblasts.

## Methods

### Cells and cell culture

We purchased SAS, a human tongue squamous cell carcinoma cell line, from the RIKEN Cell Bank (Tsukuba, Japan). FaDu, the cells from a human hypopharyngeal SCC cell line, were kindly gifted by the Department of Cell Biology and Morphology, Akita University Graduate School of Medicine (Akita, Japan). SF-TY, a human skin fibroblast cell line, was obtained from the Japanese Collection of Research Bioresources Cell Bank (JCRB Cell Bank, Osaka, Japan).

All cells were maintained in the Dulbecco’s modified Eagle’s medium (DMEM; Merck KGaA, Darmstadt, Germany) supplemented with 10% fetal bovine serum in a humidified atmosphere containing 5% CO_2_ at 37°C. For neutralization of IL-6, neutralizing mouse anti-IL-6 mAb (MAB406) and isotype control immunoglobulin G1 (IgG1) mAb (MAB005) were purchased from R & D Systems (Minneapolis, MN)

### Irradiation

Cells were seeded in 60 mm dishes and cultured to 80% confluence before being irradiated at 2, 5, or 10 Gy at room temperature using a 160 kVp cabinet X-ray system filtered with 0.5 mm Cu by the Faxitron CP-160 (Faxitron X-Ray Corp., Wheeling, IL, USA). After irradiation, cells were cultured for 24 h prior to being harvested and subjected to an IL-6 enzyme-linked immunosorbent assay (ELISA) assay or cell migration assay. To conduct a cell survival assay, cells were cultured for 6 days after irradiation before being fixed and stained with crystal violet and then being observed under a light microscope (Olympus, Tokyo, Japan).

### Cell survival assay (crystal violet staining)

The cells were fixed in 4% paraformaldehyde for 20 min at room temperature and then stained with 0.04% crystal violet in 1% ethanol (20 min at room temperature). The plates were subsequently washed extensively under running tap water and air dried. After solubilization of the samples in 1% sodium dodecyl sulfate (SDS), the optical density values were read by plate reader at 550 nm.

### Migration assay

We evaluated cell migration in vitro using semipermeable modified Boyden inserts with a pore size of 8 μm (Becton, Dickinson and Company, Franklin Lakes, NJ, USA). In total, 3 × 10^4^ SAS and FaDu cells were plated in the inserts. For the coculture assay, 5 × 10^4^ fibroblast cells were plated in a holding well. Plating was conducted on serum‑free DMEM. We plated the same number of cells from the inserts in 96-well plates to serve as loading controls. The insert contained no serum, whereas the lower well contained 10% FBS, which served as a chemoattractant. Depending on the needs of experiment, 10 ng/ml of IL-6, 20 ng/ml of IL-6 neutralizing antibody, or 20 ng/ml of control IgG were added to the medium. After 24 h of treatment at 37°C in a 5% CO_2_ incubator, we gently wiped away the cells in the insert using a cotton swab. Cells on the reverse side of the insert were fixed and stained with Diff‑Quik® (Sysmex, Kobe, Japan) according to the manufacturer’s instructions. We counted the invading cells in four representative fields using light microscopy at a magnification of 200×. Cells plated in 96-well plates were subjected to 3-(4,5-dimethylthiazol-2-yl)-2,5-diphenyltetrazolium bromide assays and we normalized the cell numbers across the groups. We also adjusted the number of migrating cells accordingly.

### Western blotting

We detected protein expression using western blot analysis with actin used as an internal control. We lysed cell lines in detergent containing 1% NP-40, 150 mmol/l NaCl, 1 mmol/l EDTA, 0.1 mmol/l phenylmethylsulfonyl fluoride, 1 μg/ml leupeptin, and 1 μg/ml aprotinin and then determined the protein levels using the Bio-Rad Protein Assay method (Bio-Rad Laboratories Inc., Hercules, CA, USA). We separated 40 μg of the total protein on 8% SDS-PAGE gels and transferred these to nitrocellulose membranes using a semidry transfer machine (Bio-Rad Laboratories). Next, we blocked membranes with 5% skimmed milk/TBS with Tween-20 solution for 1 h at room temperature, before incubating with primary antibodies in 5% skimmed milk in TBS‑T overnight at 4°C. After washing with TBS-T three times, we incubated the membranes for 1 h with horseradish-peroxidase-conjugated secondary antibody (Bio-Rad Laboratories) at 1:3,000 diluted in 5% skimmed milk in TBS‑T. We then rinsed the filters with TBS‑T three times and developed the blot using Luminol Reagent (Santa Cruz Biotechnology, Santa Cruz, CA, USA) by autoradiography. The band intensities were analyzed using ImageJ (U. S. National Institutes of Health). We used the following primary antibodies: mouse anti-IL-6Rα (1:1,000; Santa Cruz Biotechnology, Santa Cruz, USA), mouse anti-gp130 (1:1,000; Santa Cruz Biotechnology), and mouse anti-actin (1:3,000; Santa Cruz Biotechnology).

### ELISA

The serum levels of IL-6 were measured by an ELISA using an ELISA kit (Proteintech, Rosemont, IL, USA.). To compare the IL-6 production by fibroblast with SAS and FaDu under serum-free conditions, 3 × 10^6^ cells of each type were seeded on 6-well plates in DMEM containing 10% fetal bovine serum (FBS) overnight. Subsequently, the medium was replaced with serum-free DMEM and cells were cultured for 24 h. Similarly, to evaluate changes in IL-6 expression by irradiation in fibroblasts, 3 × 10^6^ fibroblasts were seeded on 6-well plates in DMEM with 10% FBS overnight. The medium was changed to serum-free DMEM and irradiated at 10 Gy. Nonirradiated fibroblasts were also prepared as a control. At 24 h after irradiation, supernatants were collected, and subjected to an ELISA assay.

### Statistical analysis

Statistical analyses were performed using Statcel 3 (OMS Publishing, Tokorozawa, Japan). A Wilcoxon–Mann–Whitney two-tailed exact test was used to assess the statistically significant differences in cell survival, migration studies, and IL-6 expression. Data are presented as means ± standard deviation (SD) from experiments that were repeated at least three times. We considered differences with P < 0.05 as statistically significant.

## Results

### Fibroblast resistance to irradiation compared with HNSCC cells

**Fig 1:**
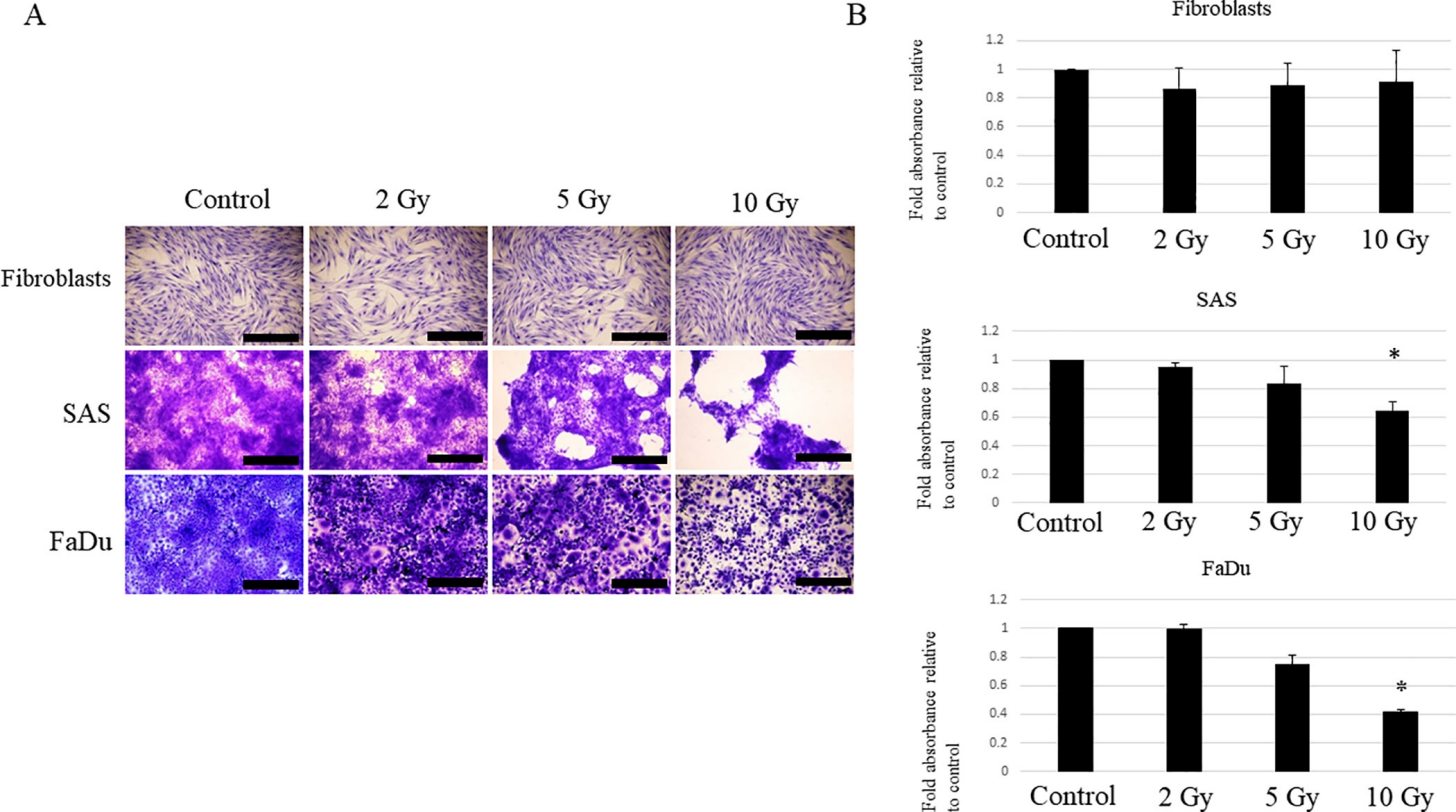
Effect of irradiation on the survival of HNSCC cells and fibroblasts. The viability of HNSCC cells and fibroblasts was evaluated by crystal violet staining. Each cell line was cultured to 80% confluence and irradiated at 2, 5, or 10 Gy. At 6 days post-irradiation, the cells were fixed and stained by crystal violet. An unirradiated condition was used as a control in each cell line. Experiments were repeated three times. (A) Representative findings of cells observed with a light microscope. Scale bar: 1000 μm. (B) The cells were solubilized by 1% SDS and absorbance was measured at 550 nm. Results are presented as fold-changes in absorbance relative to those under control conditions. Each experiment was repeated in triplicate and the data shown are means of three measurements with SD error bars. * P < 0.05.

Radiotherapy utilizes the difference in radiosensitivity between target cancer cells and healthy cells; during radiotherapy, cancer cells are killed by irradiation, whereas other cells in the surrounding microenvironment survive. In particular, the major cells in the microenvironment, fibroblasts, survive and are involved in tissue remodeling after radiotherapy [12]. In the present study, we first assessed whether there was a difference in radiosensitivity between HNSCC cells and fibroblasts. Note that two HNSCC cell lines with different origins were used in the present study for the purpose of confirming whether the observed phenomenon is common to HNSCC. When two HNSCC cell lines, SAS and FaDu, as well as a healthy fibroblast cell line were irradiated with 2, 5, and 10 Gy the survival rate of HNSCC cells decreased with irradiation in a dose-dependent manner relative to the survival of cells under nonirradiated conditions; it contrast, the fibroblasts did not show a significant decrease in survival rate (Fig 1). This result suggests that fibroblasts are more resistant to radiation than HNSCC cells and may survive even after radiotherapy.

### Irradiation enhances the ability of fibroblasts to promote HNSCC cell migration

**Fig 2:**
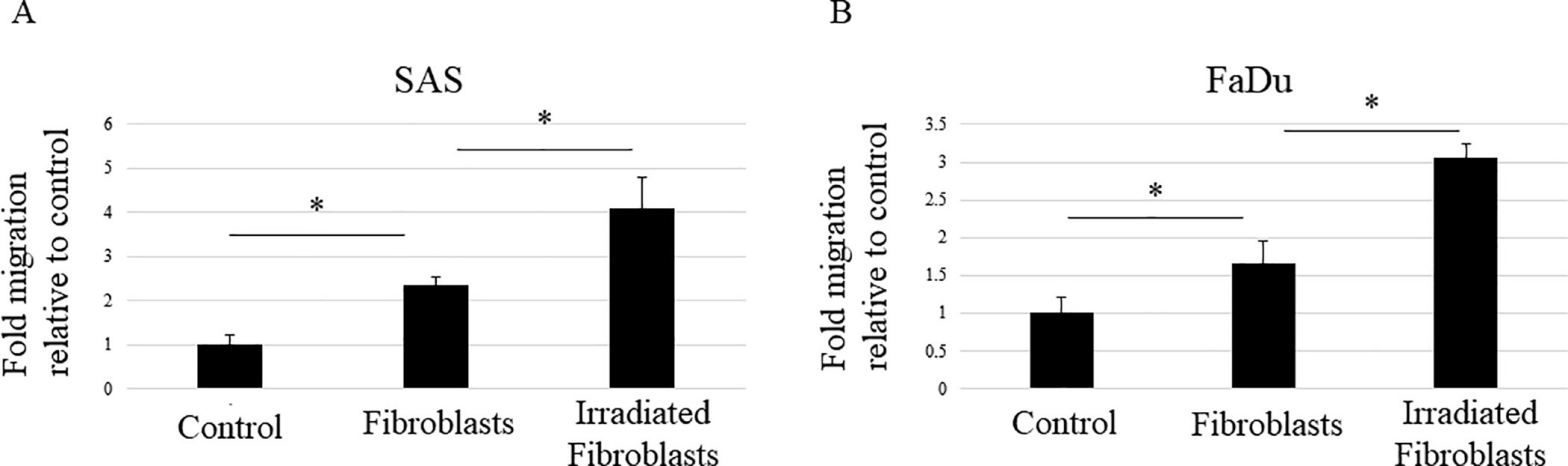
Irradiation enhances the ability of fibroblasts to promote HNSCC migration. A cell migration assay was performed with the HNSCC cell lines SAS (A) and FaDu (B). HNSCC cells were seeded into semipermeable inserts and cocultured with nonirradiated or irradiated fibroblasts seeded into the lower chamber. Results are expressed as fold-changes relative to HNSCC cells without coculture with fibroblasts (Control). HNSCC cell migration increased in the coculture with nonirradiated fibroblasts and was further increased by coculture with irradiated fibroblasts. The experiment was repeated three times and the data represent the means of three measurements with SD error bars. * P < 0.05.

Fibroblasts are known to contribute to cancer progression and to be important factors in cancer control in various solid cancers [13]. In addition, fibroblasts that survive after radiotherapy serve as a scaffold and contribute to cancer recurrence and metastasis [5, 6, 14]. In other words, irradiation enhances the ability of fibroblasts to promote the activity of cancer cells. In the present study, we investigated how irradiation affects the HNSCC-activating effect of fibroblasts. Although tumor progression is a multistep process, irradiation particularly affects tumor cell migration [3, 4]. Therefore, we focused on cell migration ability in the present study. In a migration assay, HNSCC cells were seeded into semipermeable inserts and cocultured with or without irradiated or nonirradiated fibroblasts, which were seeded into the lower chamber. Our aim was to determine whether fibroblasts influence the migration of HNSCC cells and, if so, how irradiation might affect this ability. As shown in Fig 2, the migration of HNSCC cells was enhanced even when they were cultured with nonirradiated fibroblasts, but this migration ability was further enhanced when cocultured with irradiated fibroblasts. Thus, irradiation seems to extend the ability of fibroblasts to promote HNSCC cell migration. Furthermore, the result suggests that the factor promoting the migration of HNSCC cells might be a liquid component secreted from fibroblasts.

### Irradiation increases the expression of IL-6 from fibroblasts

**Fig 3:**
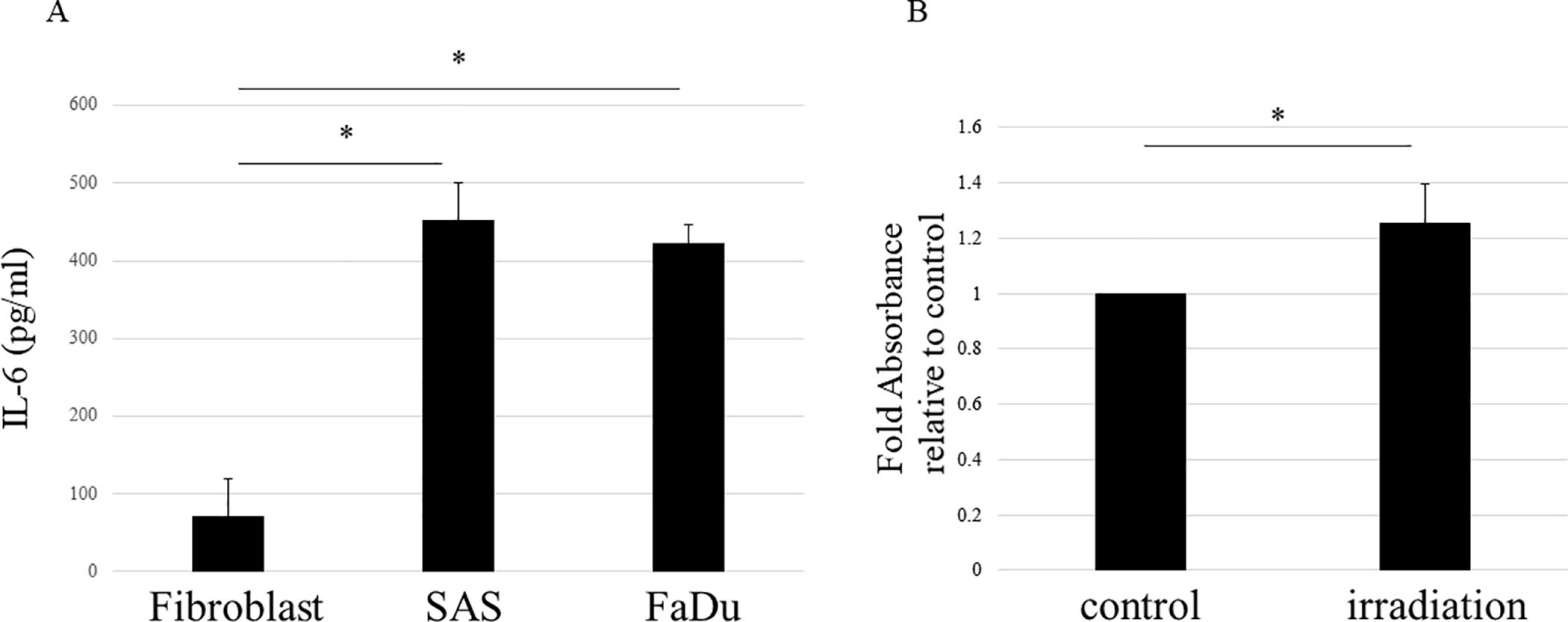
Irradiation increases expression of IL-6 from fibroblasts. The expression of IL-6 in the culture media of fibroblast and HNSCC cell lines (SAS and FaDu) were evaluated by ELISA. (A) Significantly higher levels of IL-6 were observed in the serum-free culture media of SAS and FaDu cell lines compared with fibroblasts. (B) Expression of IL-6 in culture media of fibroblasts with or without 10 Gy of irradiation was evaluated by ELISA. Results are expressed as fold-changes relative to fibroblasts without irradiation. Expression of IL-6 from fibroblasts was significantly increased with irradiation. The experiments were repeated in triplicate and the data represent the means of three measurements with SD error bars. * P < 0.05.

The results shown in Fig 2 suggest that fibroblasts use some humoral factor to induce migration of HNSCC cells and that the expression of this factor is increased by irradiation. Although various humoral factors are known to be involved in cancer progression, IL-6 has recently been associated with various carcinomas including HNSCC. Because IL-6 elicits several tumor growth-promoting effects including promotion of cancer cell migration, it is also a promising therapeutic target [15, 16]. Therefore, we investigated IL-6 as the potential factor secreted from fibroblasts; specifically, we assessed whether IL-6 secretion was increased by irradiation. According to an ELISA, we first evaluated the expression of IL-6 in the culture media of fibroblast and HNSCC cell lines, SAS and FaDu. The results showed that IL-6 expression was lower in fibroblasts compared with HNSCC cells (Fig 3A). Subsequently, we examined the effect of irradiation on the expression of IL-6 in fibroblasts. We found that the expression of IL-6 was increased in the culture media of irradiated (10 Gy) fibroblasts compared with its expression in media from nonirradiated fibroblasts (Fig 3B). These results indicate that IL-6 secretion from fibroblasts is normally lower than that detected in HNSCC cells, whereas irradiation increases IL-6 secretion from fibroblasts; it is also suggested that IL-6 plays a role in the migration of HNSCC cells triggered by fibroblast irradiation (see Fig 2).

### IL-6 induces HNSCC cell migration

**Fig 4:**
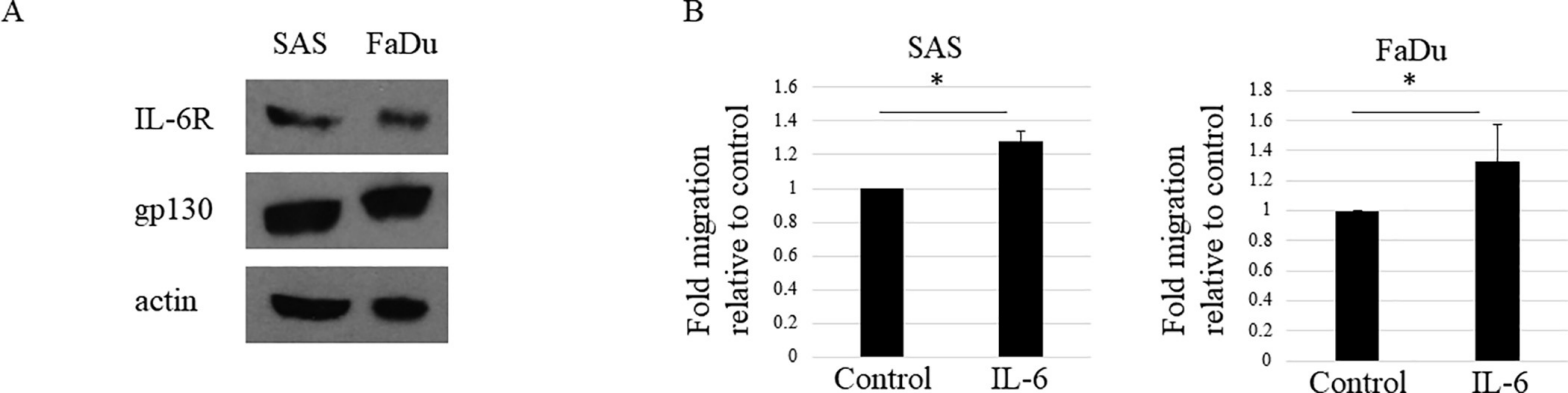
IL-6 induces HNSCC cell migration. (A) HNSCC cells express IL-6R and gp130. Expression of IL-6R and gp130 determined by western blot analysis in the HNSCC cell lines SAS and FaDu. The experiment was repeated three times with similar results. (B) IL-6 increases HNSCC cell migration. HNSCC cell lines, SAS, and FaDu, were subjected to a cell migration assay with or without 10 ng/ml of IL-6. HNSCC cell migration increased significantly with the addition of IL-6. The results are expressed as fold-changes relative to HNSCC cells without IL-6. Each experiment was repeated three times and the data shown are means of three measurements with SD error bars. * P < 0.05.

To determine whether IL-6 induces migration of HNSCC cells, we first confirmed whether HNSCC cells can be activated by IL-6. For IL-6 to demonstrate its biological activities, the target cell must express IL-6 receptor (IL-6R) and gp130 [17]. We confirmed that both SAS and FaDu HNSCC cell lines express IL-6R and gp130 via western blotting (Fig 4A). This result suggests that HNSCC cells can be activated by an intracellular signal transduction pathway via exogenous IL-6. In addition, we conducted a migration assay using HNSCC cells with or without IL-6 and found that HNSCC cell migration was increased in the presence of IL-6 (Fig 4B).

### IL-6 plays a crucial role in the ability of fibroblasts to promote HNSCC migration

**Fig 5:**
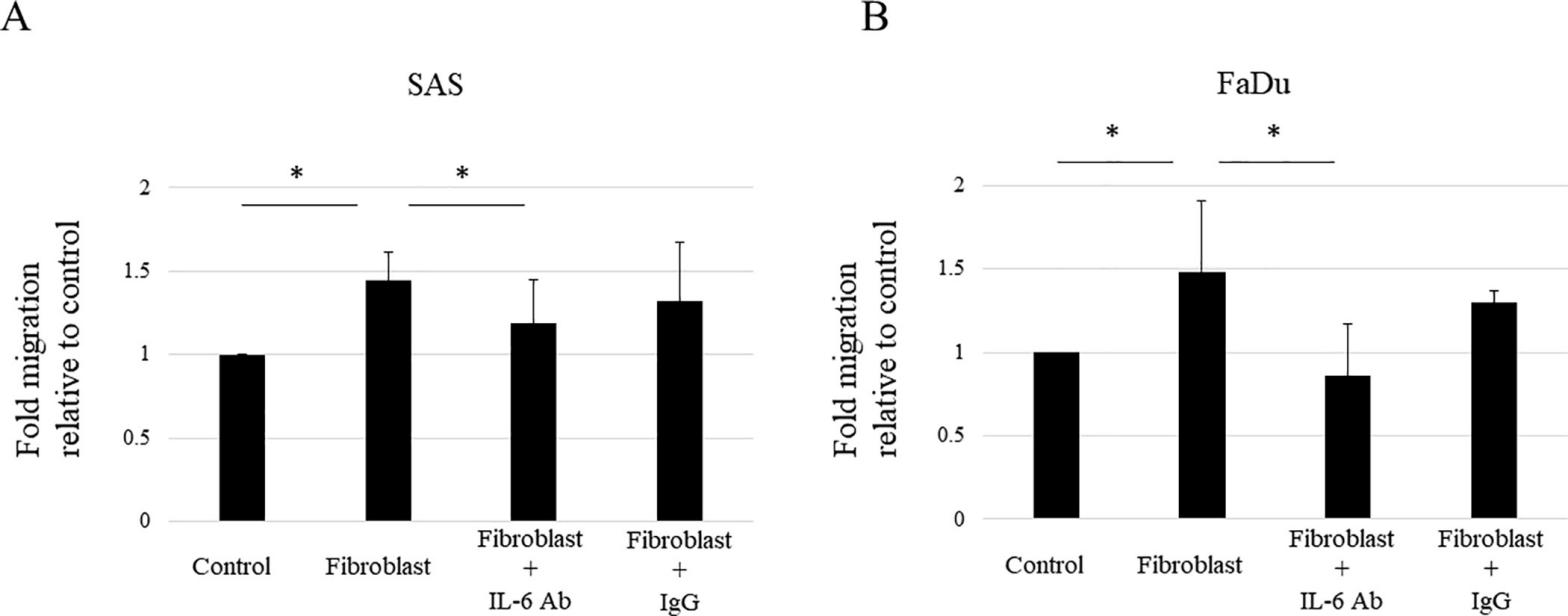
IL-6 plays a crucial role in the ability of fibroblasts to promote HNSCC migration. Neutralization of IL-6 abolishes the ability of fibroblasts to promote HNSCC migration. A cell migration assay of HNSCC cells, i.e., SAS (A) and FaDu (B), was performed with or without coculture with fibroblasts. In addition, cells were treated with 20 ng/ml of control monoclonal IgG (IgG) or anti-IL-6 mAb (IL-6 Ab) under coculture conditions. The results are expressed as fold-changes relative to HNSCC cells without coculture with fibroblasts. HNSCC cell migration increases by coculture with fibroblasts; however, this increase was prevented by treatment with anti-IL-6 mAb. Each experiment was repeated in triplicate and the data represent the means of three measurements with SD error bars. * P < 0.05.

We confirmed whether fibroblasts promote HNSCC cell migration using IL-6 secretion. For this purpose, we performed a HNSCC cell migration assay including coculturing with fibroblasts and with or without an IL-6 neutralizing antibody. Results showed that HNSCC cell migration was induced by coculturing with fibroblasts; however, this induction of HNSCC migration was significantly suppressed by the IL-6 neutralizing antibody (Fig 5). Thus, IL-6 secreted from fibroblasts is apparently involved in the enhancement of HNSCC cell migration.

## Discussion

Invasion and distant metastasis are major characteristics of cancer that are associated with the progression of the disease and poor prognosis [18]. This is true for HNSCC, especially of distant metastases that are difficult to treat despite the development of therapeutic agents [19–21]. Thus, metastasis is clinically important in cancer treatment. It has been reported that the migration of cancer cells is essential in the process of metastasis. When cancer cells invade surrounding tissues, the cells migrate between tissues while destroying the surrounding tissues with enzymes. The migrating cells then infiltrate the vessels and become tumor cells that circulate throughout the body, creating metastatic lesions at distant locations. Thus, cell migration is an important first step in distant metastasis [22].

Previous studies have shown that irradiation contributes to increased distant metastases in many cancers, including head and neck cancers [23–25]. Therefore, the enhancement of cell migration by irradiation has attracted the attention of researchers. Various studies have shown hypoxia, epithelial-to-mesenchymal transition, and specific cytokines as causes of irradiation-induced migration enhancement [3, 4]. In particular, cytokines are secreted not only from cancer cells but also from the cells in the environment surrounding the cancer, and they are important for various tumor progression phenomena such as cancer cell invasion and the increase in circulating tumor cells [8, 10]. Fibroblasts in the cancer microenvironment are known as cancer-associated fibroblasts; these are strongly associated with cancer cells and contribute to cancer progression including the progression of head and neck cancers [26, 27]. Under irradiation, fibroblasts are also important for cancer progression [28]. For example, in pancreatic and liver cancers under irradiation, fibroblasts increase the secretion of various humoral factors including cytokines such as bFGF, TNF-a, VEGF, IL-6, EGF, MMP-2, and MMP-9. These humoral factors contribute to cancer progression, including the migration and invasion of cancer cells after irradiation [7, 9]. Thus, cytokines induced from fibroblasts by irradiation have important effects on the enhanced migration ability of cancer cells after irradiation.

In the present study, we showed that IL-6 secreted from fibroblasts is an important factor in the induction of HNSCC cell migration. IL-6 has been primarily reported as a factor in inflammation, but it is also known to be involved in tumor progression in many cancers [29, 30]. Therefore, its potential as a therapeutic target for cancer has been indicated; furthermore, the antitumor effect of inhibiting the JAK/STAT3 pathway, which is the main signaling pathway of IL-6, has been reported [30, 31]. In addition, IL-6/JAK/STAT3 pathway inhibition has a synergistic antitumor effect with conventional cancer treatments, e.g., its effect is enhanced in combination with chemotherapy and radiotherapy [30, 32]. In particular, recent studies have reported that IL-6 is associated with the effects of immune checkpoint inhibitor in the treatment of head and neck cancer. Consequently, IL-6 is a factor expected to become increasingly important in the treatment of HNSCC in the future. However, it should be noted that the drawback of the current study’s design was that it did not completely focus on physiological conditions. The fibroblasts used in this study were not actual fibroblasts from the tumor stroma; however, they were normal skin-derived fibroblasts. Moreover, the experiments were not based on a three-dimensional model, and the conditions were different from *in vivo* conditions. Therefore, the results obtained in this study only allow us to speculate on the effect of healthy fibroblasts on HNSCC cells, rather than the actual changes in the interaction between HNSCC cells and the tumor stroma triggered by irradiation. Whether IL-6 is truly important as a therapeutic target for HNSCC requires careful examination under conditions that are more similar to the *in vivo* situation.

## Conclusion

The results of this study showed that fibroblasts survive irradiation and that irradiation induces expression of IL-6 from fibroblasts. It was also found that irradiation enhances the ability of fibroblasts to promote the migration of HNSCC cells.

Moreover, it was confirmed that fibroblasts induce HNSCC cell migration through IL-6 expression. Taken together, these results suggest the possibility that fibroblasts survive irradiation and enhance the promotion of HNSCC cell migration through IL-6 secretion. To the best of our knowledge, we are the first to speculate on the mechanism by which irradiation promotes HNSCC cell migration. Thus, our results could contribute to the future development of therapeutics in HNSCC including the treatment of recurrence and metastasis after radiotherapy.

## Supporting information

S1 File Raw images of western blot analyses. In addition to SAS and FaDu cells, the HSC-3 tongue cancer cell line was also included in this work (the rightmost lane). However, the HSC-3 cell line was not used in other experiments in the present study; thus, it was excluded from the drawing of Fig 4A. The part that was used in the figure is shown in a red frame.
(PDF)

## Abbreviations

DMEM: Dulbecco’s modified Eagle’s medium
ELISA: Enzyme-linked immunosorbent assay
FBS: Fetal bovine serum
HNSCC: Head and neck squamous cell carcinoma
SD: Standard deviation
SDS: Sodium dodecyl sulfate
