# Supplementary material for: Irradiated fibroblasts increase interleukin-6 expression and induce migration of head and neck squamous cell carcinoma
Source: PLoS One. 2022 Jan 28;17(1):e0262549. doi: 10.1371/journal.pone.0262549 (PMC8797190; doi:10.1371/journal.pone.0262549)
Supplement: S1 File — In addition to SAS and FaDu cells, the HSC-3 tongue cancer cell line was also included in this work (the rightmost lane). However, the HSC-3 cell line was not used in other experiments in the present study; thus, it was excluded from the drawing of Fig 4A. The part that was used in the figure is shown in a red frame. (PDF) [file pone.0262549.s001.pdf]

Figure 4A

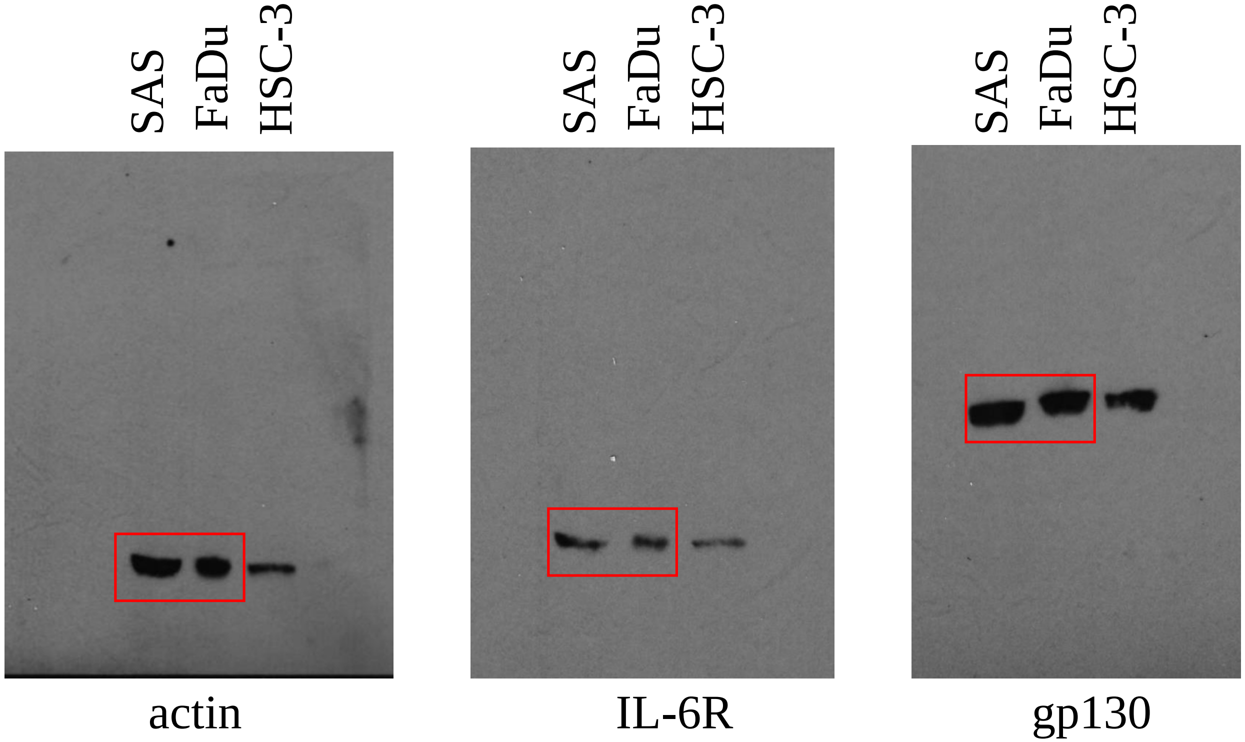

In addition to SAS and FaDu cells, the HSC-3 tongue cancer cell line was also included in this work (the rightmost lane). However, the HSC-3 cell line was not used in other experiments in the present study; thus, it was excluded from the drawing of Figure 4A. The part that was used in the figure is shown in a red frame.
